# Antidermatophytic Properties of *Ar*-Turmerone, Turmeric Oil, and *Curcuma longa* Preparations

**DOI:** 10.1155/2013/250597

**Published:** 2013-08-26

**Authors:** Mukda Jankasem, Mansuang Wuthi-udomlert, Wandee Gritsanapan

**Affiliations:** ^1^Department of Pharmacognosy, Faculty of Pharmacy, Mahidol University, 447 Sri-Ayudthaya Road, Ratchathewi, Bangkok 10400, Thailand; ^2^Department of Microbiology, Faculty of Pharmacy, Mahidol University, 447 Sri-Ayudthaya Road, Ratchathewi, Bangkok 10400, Thailand

## Abstract

*Curcuma longa* L. or turmeric of the family Zingiberaceae is widely used in Thai traditional medicines for the treatment of rash, itching, tinea, and ringworm. Previous studies on turmeric oil reported effective antifungal activity against dermatophytes, a group of fungi that causes skin diseases. In this study, turmeric creams containing 6 and 10% w/w turmeric oil were prepared and tested against clinical strains of dermatophytes using broth dilution technique. Minimum fungicidal concentrations of 6 and 10% w/w turmeric creams were found to be 312 **μ**g/mL. *Ar*-turmerone, a major compound separated from turmeric oil, promoted more effective antidermatophytic activity with the MICs of 1.56–6.25 **μ**g/mL, compared to 3.90–7.81 **μ**g/mL of standard ketoconazole. The results indicated that 6% w/w turmeric oil in the cream was suitable to be formulated as antidermatophytic preparation. Further research should be done on long-term chemical and antifungal stabilities of the preparation.

## 1. Introduction

Dermatophytosis (tinea or ringworm) is a prevalent form of fungal infections found in Thailand because of the tropical climate. The infection is caused by a group of keratinophilic fungi called dermatophytes. Fungi commonly involved are of the genera *Trichophyton, Microsporum*, and *Epidermophyton.* Dermatophytosis can transfer from soil and animals to humans and cause infection on many parts of the body [1, 2]. Historically, Thai traditional medicines containing *Curcuma longa* have been used for the treatment of dermatophytes. The dry powdered rhizome is mixed with a small volume of water and the mixture is applied onto infected skin [3].

*C. longa* L. or turmeric is a well-known medicinal plant of the family Zingiberaceae [4]. It has been used for the treatment of skin diseases, rash, itching, tinea and ringworm. Turmeric is a perennial herb with thick and ellipsoid-ovate rhizome with orange cortex inside. It is cultivated easily, wildly distributed in Thailand and other tropical and subtropical countries, and is also famous as a spice and coloring agent [5]. Medicinal uses of the rhizomes arise from volatile oil as a carminative and antifungal activity and yellow curcuminoids for antioxidative and anti-inflammatory properties. Active constituents in turmeric volatile oil are turmerone, atlantone, and zingiberone [6, 7]. Turmeric oil isolated from turmeric rhizome possesses effective antifungal activity against dermatophytes [8, 9]. The creams containing 3–8% w/w turmeric oil showed similar antidermatophytic activity [10]. 

To determine a suitable concentration of turmeric oil in a cream preparation, turmeric creams containing 6 and 10% w/w of turmeric oil were formulated and determined in this study for their antidermatophytic activities. The activities of turmeric oil and *ar*-turmerone, a major compound in the oil, were also determined and compared. The results will be useful for the development of appropriate turmeric cream as an alternative antidermatophytotic preparation.

## 2. Materials and Methods

### 2.1. Plant Materials

Turmeric rhizomes were purchased from a local market in Bangkok in April 2009. The sample was identified by Dr. Wandee Gritsanapan, and the voucher specimen (WCL0409) was kept at Department of Pharmacognosy, Faculty of Pharmacy, Mahidol University. The fresh rhizomes were cleaned and sliced into small pieces, dried in a hot air oven at 60–80°C for 48 hours, and then ground into moderate powder. Turmeric dried powder was extracted by water distillation for 60 hours to obtain volatile turmeric oil which was stored in a closed container, protected from light in a refrigerator (4°C) until used.

### 2.2. Extraction and Separation of *Ar*-Turmerone from Turmeric Oil

*Ar-*turmerone was separated from turmeric oil by preparative TLC (silica gel 60 GF_254_, hexane : ethyl acetate 97 : 3). The separated compound was purified and identified by GC-MS [11].

### 2.3. Preparation of Turmeric Cream

Oil in water turmeric creams was prepared by two-phase system containing 6 and 10% w/w turmeric oil. Both creams were separately kept in foam tubes protected from light until used.

### 2.4. Antifungal Activity Assays

#### 2.4.1. Inoculum Preparation

Clinical strains (provided by Institute of Dermatology, Bangkok) of *T. mentagrophytes*, *T. rubrum*, *E. floccosum*, and *M. gypseum* were isolated, cultured on Sabouraud dextrose agar (Himedia, Mumbai, India), and incubated at room temperature for 7–10 days to obtain active growing cultures.

#### 2.4.2. Determination of Minimum Inhibitory Concentration (MIC)

Turmeric oil, *Ar*-turmerone, and 6% and 10% w/w turmeric creams were tested for their antifungal susceptibility by broth microdilution assay in accordance with the Clinical and Laboratory Standard Institute guideline in the M38-A9 document on filamentous fungi [12] with some modifications. Briefly, in a U-shaped 96-well microliter plate (Thermo Fisher Scientific, NY, USA), each well was filled with 100 *μ*L of twofold serially diluted sample and 100 *μ*L of spore suspension of each tested organism. The final concentration of inoculum was adjusted to 0.4 to 5 × 10^4^ CFU/mL. Each sample was done in triplicate, while growth control of each strain was filled with 200 *μ*L of the inoculums. Cream base was used as a negative control, while ketoconazole and miconazole nitrate cream solution (Daktarin, 70 mg/mL) were used as positive controls. The MICs, the lowest concentration of the sample that shows no visible growth, after incubation at 28°C for approximately 72–96 hours (or indicated by growth control as the end point) of turmeric oil, *ar*-turmerone, 6% and 10% w/w turmeric creams, ketoconazole, and Daktarin cream solution were determined. The minimum fungicidal concentrations (MFCs), defined as the lowest concentration that prevent the fungal growth on the solid medium, were also investigated by subculturing the MICs of each sample onto a new SDA plate and incubated at 28°C for approximately 72–96 hours or indicated by growth control as the end point.

## 3. Results and Discussion

By water distillation, the yield of turmeric oil from the dried rhizomes was 6.3% v/w. MICs of turmeric oil and cream base against all tested dermatophytes were 1.56–6.25 and 2500 *μ*g/mL, respectively. Turmeric oil possesses higher antifungal activity than cream base, indicative of the effective and active constituent in turmeric cream preparations. MICs of 6 and 10% w/w turmeric creams were found to be 78–312 and 78–156 *μ*g/mL, respectively while MICs of Daktarin cream solution was 54.68 *μ*g/mL. MICs of 10% w/w turmeric cream proved more effective than the 6% w/w cream, but MFCs of the both creams expressed relatively equal activity (Table 1). The major compound in turmeric oil, identified as *Ar*-turmerone, promoted effective antidermatophytic activity against all dermatophytes with MICs of 3.90–7.81 *μ*g/mL, which is lower than the MIC values of standard ketoconazole at 6.25–25 *μ*g/mL as shown in Table 1. 

This study reveals that turmeric creams exhibits antifungal activity and is effective against dermatophytes. This is confirmative of the ethnopharmacological use of this medical plant to treat skin diseases, especially tinea and ringworm. As a result, the 6% w/w turmeric cream is suitable as an alternative antidermatophytic preparation and could be subjected to further development. 

## 4. Conclusions

*Ar*-turmerone, a major compound in turmeric oil, showed effective antidermatophytic activity. It could be used as an active marker for quality assessment of turmeric oil and active ingredient in turmeric creams and other finished antifungal products. The turmeric cream with 6% w/w turmeric oil was suitable for further development as an alternative antidermatophytic preparation. All samples have effective antifungal activity against tested dermatophytes, especially against *T. rubrum*, a common dermatophyte which is widely spread throughout the world. However, further research on long-term physical, chemical and antifungal activity stabilities of the turmeric cream should be done to find the optimum storage condition. The clinical trial of the cream could also be tested on the affected patients to explore the practicality of the cream and the possibility of commercial development.

## Figures and Tables

**Table 1:** Average MICs and MFCs of turmeric oil, *Ar*-turmerone, 6% and 10 %w/w turmeric creams, cream base, ketoconazole, and Daktarin cream solution.

Tested samples	MICs (*μ*g/mL)				MFCs (*μ*g/mL)			
	*T. rubrum *	*T. mentagrophytes *	*M. gypseum *	*E. floccosum *	*T. rubrum *	*T. mentagrophytes *	*M. gypseum *	*E. floccosum *
Turmeric oil	1.56	6.25	6.25	1.56	3.12	6.25	6.25	1.56
*Ar*-turmerone	3.90	7.81	7.81	3.90	62	125	62	31.25
6 %w/w turmeric cream	156	312	312	78	312	312	312	156
10 %w/w turmeric cream	78	156	156	78	312	312	312	78
Cream base	2500	2500	2500	2500	2500	2500	2500	2500
Ketoconazole	6.25	12.50	25	12.50	25	12.50	25	6.25
Daktarin	54.68	54.68	54.68	54.68	54.68	54.68	54.68	54.68

## References

[1] Greenwood D (2007). *Microbiology, A Guide To Microbiology Infections: Pathogenesis, Immunity, LaboraTory Diagnosis and Control*.

[2] Rippon JW (1988). *Medical Mycology—the Pathogenic Fungi and the Pathogenic Actinomycetes*.

[3] Wutthithamavet W (1997). *Thai Traditional Medicine,”*.

[4] Smitinand T (2001). *Thai Plant Names*.

[5] Jirawongse VA (1995). *Thai Herbal Pharmacopoeia *.

[6] Leung AY, Foster S (1996). *Encyclopedia of Common Natural Ingredients Used in Food, Drugs, and Cosmetics*.

[7] Pothitirat W, Gritsanapan W (2006). Variation of bioactive components in *Curcuma longa* in Thailand. *Current Science*.

[8] Apisariyakul A, Vanittanakom N, Buddhasukh D (1995). Antifungal activity of turmeric oil extracted from *Curcuma longa* (Zingiberaceae). *Journal of Ethnopharmacology*.

[9] Wuthi-udomlert M, Grisanapan W, Luanratana O, Caichompoo W (2000). Antifungal activity of *Curcuma longa* grown in Thailand. *Southeast Asian Journal of Tropical Medicine and Public Health*.

[10] Pitakvongsaporn P (2000). *The study of antifungal activity stability and skin irritation of turmeric oil [M.S. thesis]*.

[11] Adams RP (2007). *Identification of Essential Oil Components by Gas Chromatography/ Mass Spectrometry*.

[12] Clinical (2008). *Reference Method For Broth Dilution Antifungal Susceptibility Testing of Filamentous Fungi, Approved Standard M38-A9*.

